# Exploring the Role of Mindfulness in the Well-being of Junior Doctors

**DOI:** 10.1192/bjo.2022.116

**Published:** 2022-06-20

**Authors:** Matthew Cohen, Chanais Matthews, Chris Bu, Marc Jones, Jasmine Hearn

**Affiliations:** 1 King's College London, London, United Kingdom.; 2 Manchester Metropolitan University, Manchester, United Kingdom; 3 Merseycare NHS Foundation Trust, Liverpool, United Kingdom

## Abstract

**Aims:**

This study aims to explore the relationship between mental well-being (The Warwick-Edinburgh Mental Well-being Scale), stress (Appraisal of Life Events Scale) and mindfulness (5 Facet Mindfulness Questionnaire) by means of a questionnaire.

**Methods:**

The questionnaire was part of a mixed-method study looking into Mindfulness Resilience and Effectiveness Training in foundation doctors. In total 144 foundation doctors across the North West of England completed the questionnaire over a period of 5 months.

**Results:**

A Pearson's correlation coefficient was used to assess the relationships between mental well-being, appraisal of stress and mindfulness. Results show that there was a significant, negative, and somewhat weak association between mental well-being and the appraisal of stressful life events (r = (142) –.23, p = .006). A significant, positive, and strong relationship was also found between the two variables mindfulness and mental well-being (r (142) = .60, p < 0.001), in addition to a significant, weak positive relationship between mindfulness and appraisal of stressful life events (r (142) = –.18, p = .033).

**Conclusion:**

The results indicate that those with greater mental well-being were better able to tolerate stressful life events and appraise them as a challenge. Likewise, those with greater mindfulness scores showed greater well-being suggesting that improving one may improve the other. This has implications for intervention development (e.g., training in mindfulness) which can help to further improve well-being and appraisal of stressful life events in trainee doctors.

